# Supplementary material for: Influence of Mechanical Loading on the Process of Tribochemical Action on Physicochemical and Biopharmaceutical Properties of Substances, Using Lacosamide as an Example: From Micronisation to Mechanical Activation
Source: Pharmaceutics. 2024 Jun 13;16(6):798. doi: 10.3390/pharmaceutics16060798 (PMC11207894; doi:10.3390/pharmaceutics16060798)
Supplement: Supplementary file 1 [file pharmaceutics-16-00798-s001.zip › pharmaceutics-2995989-supplementary.pdf]

Influence of Mechanical Loading on the Process of Tribochemical Action on Physicochemical and Biopharmaceutical Properties of Substances, Using Lacosamide as an Example: From Micronisation to Mechanical Activation

Elena V. Uspenskaya, Ekaterina Kuzmina, Hoang Thi Ngoc Quynh, Maria A. Komkova, Ilaha V. Kazimova and Aleksey A. Timofeev

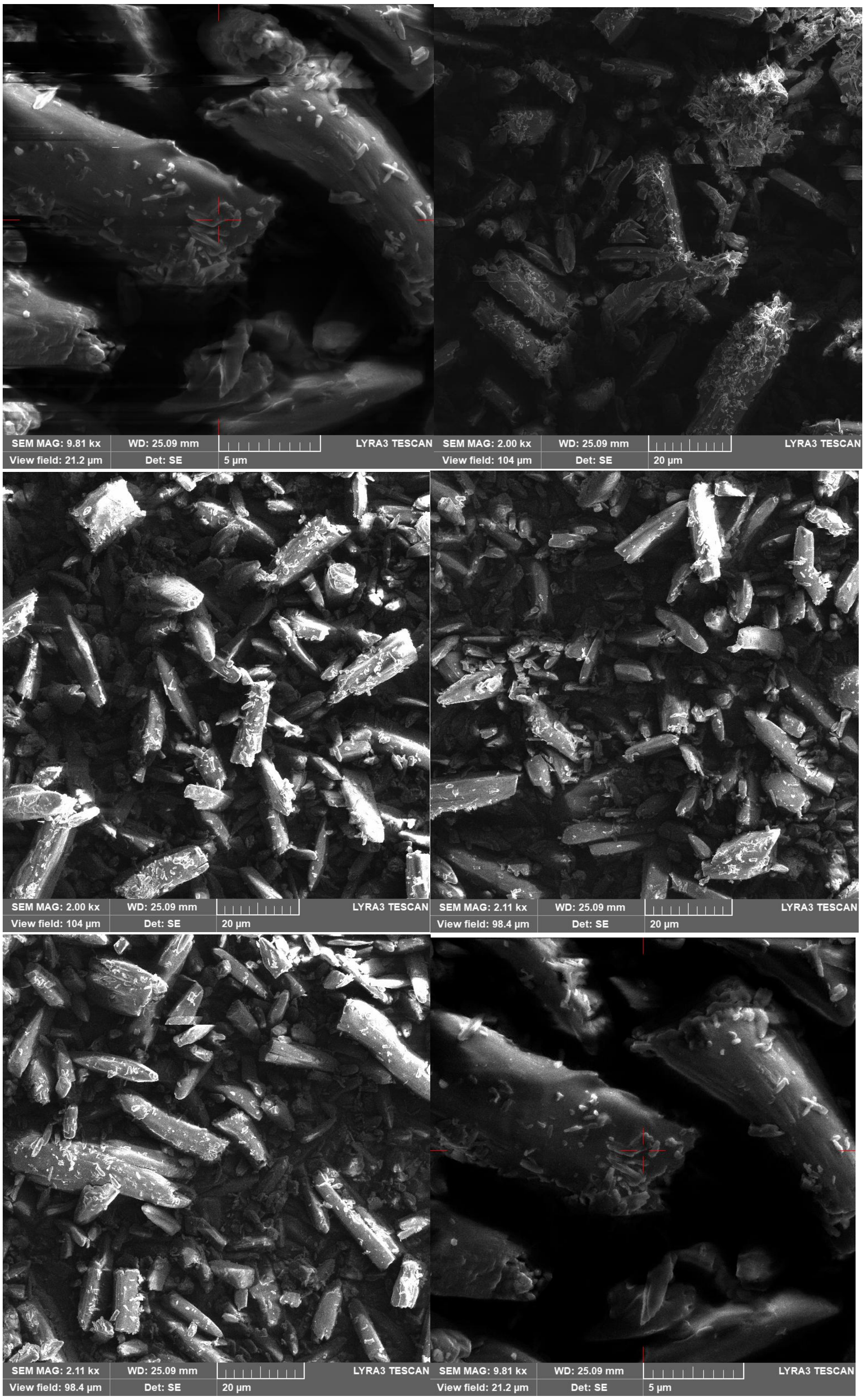

Figure S1: SEM micrographs for Lcs native obtained at t = 0 min of high-intensity ML. Device magnification (MAG) MAG = 9.81–2.11kx.

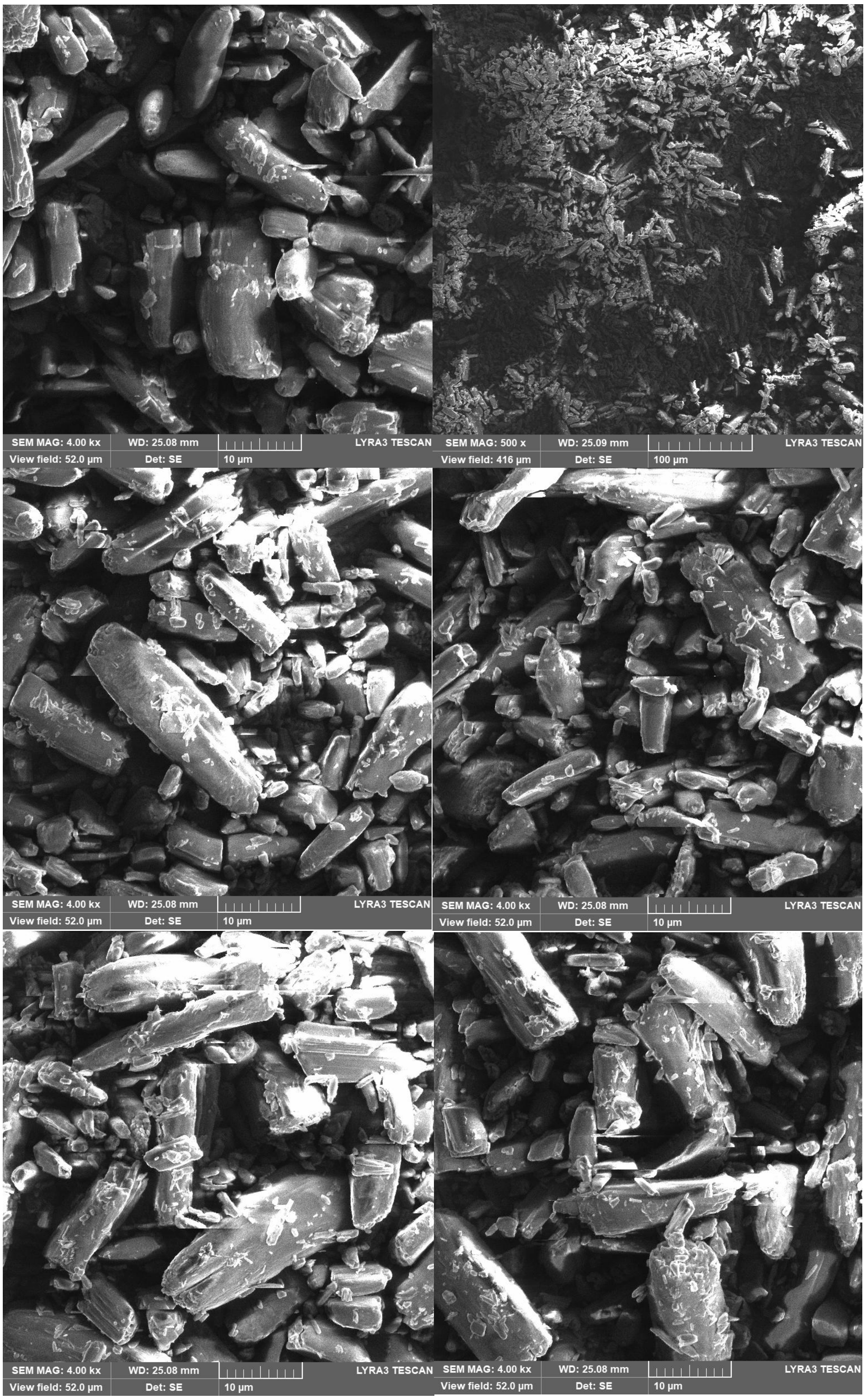

Figure S2: SEM micrographs for Lcs obtained at t = 60 min of high-intensity ML. Device magnification (MAG) MAG = 4.00kx–500x.

Supplementary Material

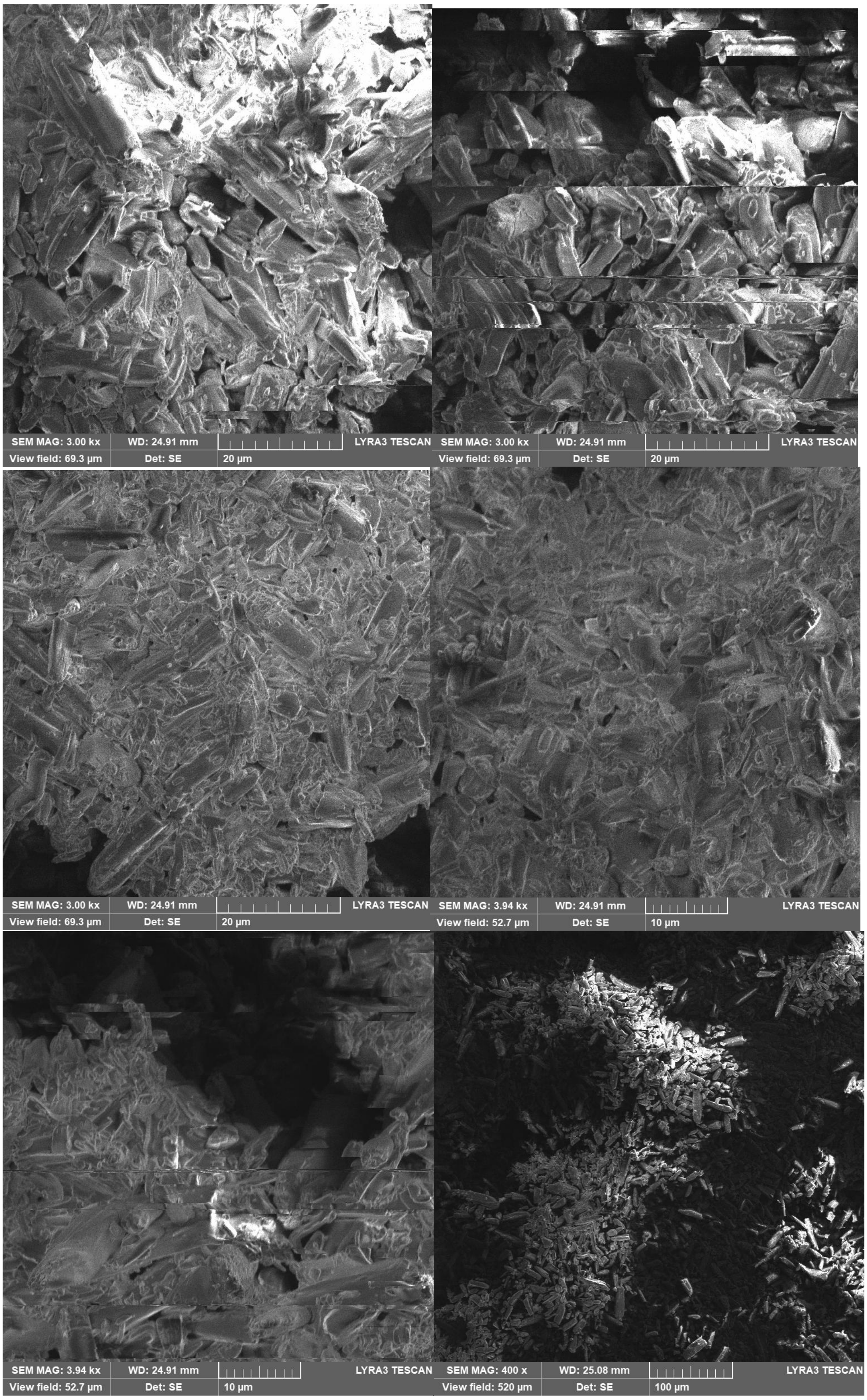

Figure S3: SEM micrographs for Lcs obtained at t = 90 min of high-intensity ML. Device magnification (MAG) MAG = 3.00kx–400x.
